# Beneficial effects of vigorous fluid resuscitation therapy varied depending on the time of onset and the sepsis stage in rats: preliminary study

**DOI:** 10.1186/cc12968

**Published:** 2013-11-05

**Authors:** Ana MA Liberatore, Ivan HJ Koh

**Affiliations:** 1 Department of Pediatric, Federal University of São Paulo, SP, Brazil; 2 Department of Surgery, Federal University of São Paulo, SP, Brazil

## Background

Fluid replacement has been a usually recommended maneuver in sepsis; however, growing clinical controversies in the management of critically ill patients with severe sepsis have questioned its benefit. Herein, we evaluated the effect of a rapid hyperhydration (HH) therapy in varying stages of sepsis.

## Materials and methods

Wistar-EPM rats, weighing 200 to 250 g, were submitted to two sepsis models: S8 group, submitted to 2 ml *Escherichia coli *10^8 ^CFU/ml intravenous (i.v.) inoculation, LD_60_, or S9 group, with *E. coli *10^9 ^CFU/ml inoculation, LD_80_. Both groups were treated with HH (30 ml/kg of Ringer lactate i.v., in 20 minutes) in the early (E30 minute) and late (L6 hour) phases of sepsis. The mortality was followed up to 30 days (*n *= 6/group) and the splanchnic microcirculation was monitored by sidestream dark field imaging (SDF) video microscopy at 6-hour and 24-hour periods (*n *= 3/group/period).

## Results

The HH at the E30 minute phase of S8 improved the survival rate from 40% to 90%, and L6 hour phase HH promoted an 80% survival rate. Besides, the survival rate in S9 (LD_80_), with E30 minute HH, improved the survival rate from 20% to 50%. However, it was less effective as compared with the E6H phase HH, which resulted in an expressive survival rate (from 20% to 70%). These intriguing results suggested that there is an interdependent and time-dependent pathophysiology feature within the host response based on sepsis severity stage and a rapid high-volume reposition. The SDF analysis in control sepsis groups (S8 and S9), without fluid therapy, showed a broadly distributed microcirculation dysfunction in the liver lobules and kidney tubules at 6 hours after sepsis challenge, and such findings were similar between groups, but after 24 hours the survivors showed an improved microcirculation hemodynamic pattern and it was more evident in the S8 group. The survivals of the S8 E30 minute treated group showed less injury at 6 hours and 24 hours as compared with nontreated groups and S8 L6 hour treated animals. In S9 treated groups, both showed a partial repair at 24 hours post sepsis.

## Conclusions

The hyperfluid therapy given rapidly in both early and late phases in sepsis and severe sepsis states showed that its beneficial effect was more or less effective dependent on the phase and sepsis intensity; however, the more prominent survival rates were seen at the early phase of sepsis (S8) and at the later phase of severe sepsis (S9). The underlying pathophysiology evolved in these paradoxical conditions needs to be better elucidated.

